# A 22.5 kb deletion in *CUL4B* causing Cabezas syndrome identified using CNV approach from WES data

**DOI:** 10.1002/ccr3.3381

**Published:** 2020-09-29

**Authors:** Maria López, Virginia Pérez‐Grijalba, Inmaculada García‐Cobaleda, Elena Domínguez‐Garrido

**Affiliations:** ^1^ Molecular Diagnostics Laboratory Fundación Rioja Salud Logroño Spain; ^2^ Unidad de Diagnóstico y Asesoramiento Genético Hospital Universitario Nuestra Sra de Candelaria Santa Cruz de Tenerife Spain

**Keywords:** Cabezas syndrome, CUL4B, intellectual disability, XLID

## Abstract

Detecting clinical grade CNV based on WES is being improved in the NGS era.

## Graphical abstract

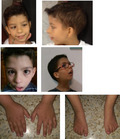

## 1 INTRODUCTION

Cabezas syndrome, which is characterized by intellectual disability, short stature, and speech delay, together with other more variable features and caused by variants in *CUL4B* gene located at Xq23. We used whole‐exome sequencing (WES) data to study copy number variation (CNV). We detected a 22.5 kb deletion in *CUL4B* gene in a 10‐year‐old boy with severe intellectual disability and clinically undiagnosed. The increasing use of NGS for undiagnosed ID cases is helping to find a genetic explanation for rare or complex disorders.

Intellectual disability (ID) includes a set of clinically and genetically heterogeneous disorders in which brain development and/or function are compromised. ID is defined by substantial limitations in both intellectual functioning and adaptive behavior with onset before the age of 18 years. According to the phenotype, ID is usually divided into two categories: nonsyndromic, in which ID is the only clinical feature, and syndromic, associated with other neurological and/or behavioral manifestations and structural anomalies. ID affects 1%‐3% of the population and is more prevalent in males than in females.^1^ This observation, along with the identification of ID families with extended X‐linked pedigrees, indicated the presence of pathogenic variants.^2^ To date, pathogenic variants in more than 100 genes have been found to cause X‐linked ID (XLID). XLID is the most common cause of ID in males and accounts for 5%‐10% of all IDs.^3^ Cabezas syndrome (MIM #300354) is a type of syndromic XLID characterized by ID, short stature, hypogonadism, and abnormal gait, together with other more variable features such as speech delay, prominent lower lip, and tremor. This syndrome is known to be caused by pathogenic variants in *CUL4B* gene (MIM #300304, NM_003588). The *CUL4B* gene is located on ChrX:120523858‐120560962 reverse strand. This gene contains 20 coding exons and encodes a 895‐amino acid protein that is a core component of the cullin‐RING‐based E3 ubiquitin protein ligase complex.^4^ *CUL4B* is thought to play significant roles in cellular processes including ubiquitination of histones and control of the cell cycle through downregulation of cyclin E and β‐catenin.

Since the description of the first family by Cabezas et al,^5^ several *CUL4B*‐associated XLID cases have been reported, sharing some of the characteristic features of the Cabezas syndrome.^3^, ^4^, ^6^, ^7^, ^8^, ^9^, ^10^, ^11^ Variants described in *CUL4B* included missense, frameshift, and primary truncations variants. They appear to be distributed throughout the gene and in most cases result in significantly reduced CUL4B protein expression.^12^ Even noncoding deletion located in 5´UTR which eliminates *CUL4B* expression has been described.^2^

Analysis by targeted exome sequencing of individuals with clinical ID, who previously tested negative for pathogenic variants and copy number variants (CNVs) in a subset of known ID related‐genes, reveals *CUL4B* as one of the most frequently mutated genes underlying XLID. More specifically, the prevalence of *CUL4B* variations in XLID has been estimated to be between 2% and 3%.^6^, ^11^ In this report, we describe the first case of Cabezas syndrome in a boy with ID and severe speech delay, caused by a 22.5 kb deletion in *CUL4B* gene detected by CNV analysis from whole‐exome sequencing (WES) data.

## 2 MATERIAL AND METHODS

**Figure 1:**
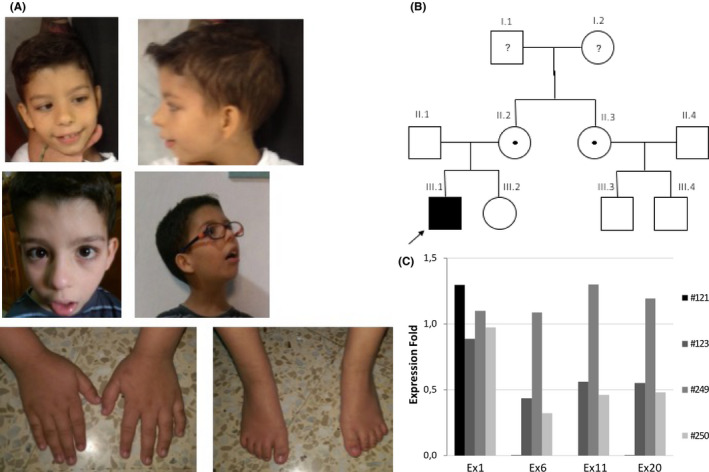
A, Patient photographs at 5 and 7 y.o., hands and feet. B, Family pedigree. C, qPCR results of exons 1, 6, 11, 20 located in *CUL4B* gene (#121 proband, #123 mother, #249 sister. #250 aunt)

Proband was a 10‐year‐old boy, first‐born child of healthy, nonconsanguineous parents. The mother had a previous miscarriage. He was delivered at full term with a birth height of 49 cm, weight of 2.815 g, and plagiocephaly. Psychomotor development was delayed: He holds his head up at the age of 7 m.o., he sat at 1 y.o., and he never crawled. He started to walk between 1.5 and 2 y.o. He showed severe intellectual disability, pronounced language delay: He is not able to speak a single word. He suffered several behavioural problems such as anxiety, autism/autism‐like conduct, and aggressive and self‐injurious character. He had short stature, slight macrocephaly, low set ears, nasal rounded tip, strabismus, prominent lower lip, dental crowding, small feet, and broad toes (Figure 1A). He also suffered seizures, mild sensorineural hearing loss, gait abnormality, fine motor delay, and wasting lower leg muscles. First clinical suspicion was Rubinstein‐Taybi syndrome (RSTS).

Clinical data, samples, and photographs were obtained after written informed consent. This work has been approved by the Committee for Ethics in Clinical Research in La Rioja (CEICLAR).

Blood samples from the proband and his parents, as well as from his sister and maternal aunt, were collected in EDTA tubes. DNA was extracted using QIAamp DNA Mini Kit (QIAGEN) following the manufacture's protocol. Following the first suspicion of RSTS, MLPA of *CREBBP* and *EP300* was performed (P313 and P333 Kit, MRC‐Holland) and panel‐based next‐generation sequencing (NGS) of *CREBBP* and *EP300* genes was carried out. Since MLPA was normal and variants were not found in this panel, WES was performed in the patient (SureSelect^XT^ Human All Exon V6, Agilent, Santa Clara, USA sequenced on an Illumina HiSeq sequencing system, coverage 100X). The resulting reads were mapped to the human genome hg19 using Burrows‐Wheeler Aligner (BWA). Sequence variants were called using the Genome Analysis Toolkit (GATK), and called variants were annotated with Annovar. ExAC browser of Broad Institute, 1000 Genomes database, and dbSNP138, as well as the Human Gene Mutation Database (HGMD), Leiden Open Variation Database (LOVD), and ClinVar databases, were checked to assess the presence/absence of detected alterations in variation repositories. A second analysis was performed to calculate CNVs with CNVkit ^13^ and detected CNVs were corroborated by qPCR. For this purpose, primers targeting exons 1, 6, 11, and 20 were designed, and qPCR assays were performed using LightCycler 480 SYBR Green I Master (Roche). *CUL4B* primer sequences and qPCR conditions are available under request.

## 3 RESULTS AND DISCUSSION

No pathogenic variants were detected after analysis of WES results. However, CNVs assessment revealed a 22.5 kb deletion at chromosome X position chrX: g.120526646‐120546685del (GRCh38p.13) that includes exons 4‐20 of *CUL4B* gene.

As a result of this molecular finding, the patient was diagnosed with Cabezas syndrome (Figure 1C).

The common phenotype, first described by Cabezas et al,^5^ is shared by most patients and includes ID ranging from mild to severe, delayed psychomotor development, severe speech delay, ataxia gait, tremor, *pes cavus*, and seizures. Growth retardation is also a hallmark of this syndrome. After the molecular diagnosis of Cabezas syndrome in our patient, we reviewed the clinical data reveling that most of these features were present, corroborating the molecular diagnosis (Table 1): severe ID, muscle wasting, delayed growth, prominent lower lip, hypogenitalism, seizures, abnormal gait, diminished fine motor skills, and severe language deficit. No *pes cavus*, obesity, or tremor was found in our patient.

**Table 1:** Summary of clinical data in patients with *CUL4B* variants

	Cabezas et al (2000)^5^	Zou et al (2007)^4^	Tarpey et al (2007)^6^	Badura‐Stronka et al (2010)^7^	Isidor et al (2010)^8^	Ravn et al (2012)^9^	Londin et al (2014)^10^	Vulto‐van Silfhout et al (2015)^11^	Okamoto et al (2017)^3^	Weissbachet al (2017)^14^	This case	Total	
No. Affected	5	6	8 families	3	1	2	8	29	1	1			
Neurological													
Intellectual disability	5/5	6/6	22/22	3/3	1/1	2/2	8/8	24/24	1/1	0/1	+	73/74	98.65%
Motor delay	5/5	6/6	5/5	na	1/1	2/2	8/8	23/23	1/1	0/1	+	52/53	98.11%
Language/speech delay	4/5	6/6	18/18	3/3	1/1	2/2	8/8	23/23	1/1	0/1	+	67/69	97.10%
Seizures	na	4/5	8/11	0/3	0/1	2/2	1/8	7/22	1/1	0/1	+	24/55	43.64%
Tremors	4/5	1/5	11/13	2/3	1/1	2/2	na	9/20	0/1	0/1	−	30/52	57.69%
Gait disturbances	3/5	6/6	6/12	na	1/1	2/2	0/8	10/21	0/1	1/1	+	31/58	53.44%
Behavioral problems	3/5	5/6	12/15	3/3	1/1	1/2	na	13/22	0/1	na	+	39/56	69.64%
Dysmorphims													
Microcephaly	0/5	6/6	–	1/3	1/1	–	3/8	–	–	na	−	11/60	18.33%
Macrocephaly	0/5	–	8/11	–	–	2/2	–	7/22	1/1	na	−	18/60	30.00%
Prominent lower lip	4/5	5/5	6/17	2/3	1/1	2/2	na	18/23	1/1	na	+	40/58	68.96%
Low set ears	0/5	0/6	na	3/3	1/1	na	8/8	17/19	1/1	na	+	31/44	70.45%
Muscle/Skeletal													
Short stature	5/5	6/6	7/11	2/3	1/1	2/2	8/8	17/22	1/1	0/1	+	50/61	81.97%
Short feet	5/5	na	7/14	3/3	na	na	8/8	14/19	na	na	+	38/50	76.00%
Gap between 1&2 toes	3/5	na	11/13	3/3	1/1	na	8/8	na	na	na	−	26/31	83.87%
*Pes cavus*	na	0/5	7/8	0/3	1/1	2/2	na	2/11	na	na	−	12/31	38.71%
Kyphosis	4/5	na	3/18	na	1/1	na	6/8	6/17	na	0/1	+	21/51	41.18%
Muscle wasting	5/5	na	7/12	1/3	na	na	na	5/11	na	0/1	+	19/33	57.57%
Other													
Genital abnormalities	4/5	2/5	10/15	1/3	1/1	0/2	8/8	17/20	1/1	0/1	+	45/62	72.58%
Obesity	4/5	0/5	15/19	2/3	0/1	2/2	0/8	11/21	0/1	0/1	−	34/67	50.75%

Driven by the molecular finding in this patient, the presence of the deletion was also checked in some relatives by qPCR. The mother and the aunt were found to be asymptomatic carriers of the same pathogenic variant, and his sister exhibited normal doses. No Cabezas syndrome phenotype has been observed in his cousins (not studied) (Figure 1B).

As in our patient, in most reported cases, the clinical diagnosis of Cabezas syndrome was established after the identification of pathogenic variants in *CUL4B*, suggesting that Cabezas syndrome is usually underdiagnosed. In this line, some authors have developed useful guidelines to identify potential patients with *CUL4B* variants.^3^, ^11^

CUL4B is a component of the ubiquitin system. Ubiquitination is a chemical reaction whereby proteins are marked for degradation or transport to specific compartments within the cell. It is also involved in histone modification and, by extension, may play a role in gene expression. CUL4B is the first ubiquitin E3 ligase mutated in XLID. Abnormalities of E3 ligases, however, have been reported in other human genetic diseases. Pathogenic variants in the ubiquitin‐protein ligase E3A gene (*UBE3A*) underlie a subset of Angelman syndrome, in which ID is a notable clinical feature, and abnormalities in other E3 ligases have been shown to cause other diseases (recessive juvenile Parkinson disease, autoimmune polyendocrinopathy syndrome type 1, etc).^12^

All the cases of Cabezas syndrome described showed ID and speech delay. This language deficiency was evident in early childhood and remained disproportionately severe given the degree of intellectual impairment; in fact, our patient, at the age of 10, was not able to speak a single word, and in most of the cases speech was very limited or nonexistent. Notably, speech impairment is also present in other syndromes caused by defects in genes involved in ubiquitination processes, such as Angelman syndrome and the X‐linked syndromic ID type Nascimento (MIM #300860) caused by *UBE2A* gene variants. Short stature, another typical feature of Cabezas syndrome, has also been linked to anomalies of the ubiquitination, as is the case of 3‐M syndrome caused by *CUL7* pathogenic variants.^8^

*CUL4B* gene should be considered as a first approach in males with ID, speech and motor delay, and behavior abnormalities. The increasing use of NGS for undiagnosed ID cases is helping to find a genetic explanation for rare or complex disorders. The clarification of the etiological diagnosis is necessary in order to answer the questions regarding the possibilities for therapeutic intervention and the risk of recurrence.

## CONFLICT OF INTEREST

The authors declare no conflict of interest.

## AUTHORS’ CONTRIBUTIONS

ML and EDG: contributed to conception or design of the work. IGC: contributed to data collection. ML, VPG, and EDG: analyzed and interpreted the data. ML, VPG, and EDG: drafted the article. ML, VPG, IGC, and EDG: revised the article. IGC and EDG: read and approved the version to be published.

## Data Availability

The datasets used and/or analyzed during the current study are available from the corresponding author on reasonable request.
